# Levels of formaldehyde vapor released from embalmed cadavers in each dissection stage

**DOI:** 10.1007/s11356-016-6744-8

**Published:** 2016-05-06

**Authors:** Yota Sugata, Hidenobu Miyaso, Yoko Odaka, Masatoshi Komiyama, Noboru Sakamoto, Chisato Mori, Yoshiharu Matsuno

**Affiliations:** 1 Department of Bioenvironmental Medicine, Graduate School of Medicine, Chiba University, Chiba, 260-8670 Japan; 2 Center for Preventive Medical Sciences, Chiba University, Chiba, 260-8670 Japan; 3 Department of Anatomy, Tokyo Medical University, Tokyo, 160-8402 Japan; 4 Division of Living Environmental Science, Chiba Prefectural Institute of Public Health, Chiba, 260-8715 Japan; 5 Kimitsu Health and Welfare Center, Chiba, 292-0832 Japan; 6 Department of Physiology and Biochemistry, Graduate School of Nursing, Chiba University, Chiba, 260-8672 Japan

**Keywords:** Formaldehyde, Gross anatomy, Embalmed cadaver, Dissection stage, Skin incision, Adipose tissue

## Abstract

Formaldehyde (FA) is an aldehyde used in antiseptics and adhesives. The World Health Organization (WHO) and other institutes have linked FA to sick building syndrome and allergic diseases. Recent studies have reported that cadavers embalmed using formalin and ethanol-based preservative solutions release FA vapor during dissection and that FA vapor may adversely affect students and lecturers in gross anatomy laboratories. However, few details have been reported correlating dissection stage with increased FA vapor release. In this study, we evaluated the vapor level of FA released in each dissection stage. Six cadavers for which consent was given for use in anatomy research and education were examined in this study. Using an active sampling method, FA vapor was collected above the thoracoabdominal region of each dissected cadaver. FA was eluted from each sampler using acetonitrile and analyzed by high-performance liquid chromatography. Our data show that FA levels significantly increase after skin incision and that the vapor level of FA released differs between male and female cadavers. We also found that subcutaneous adipose tissues of the thoracoabdominal-region release FA vapor and that female cadavers release significantly higher levels of FA per kilogram of subcutaneous adipose tissue than do male cadavers. Based on these data, we propose the methods be developed to prevent exposure to FA vapors released from cadavers.

**Electronic supplementary material:**

The online version of this article (doi:10.1007/s11356-016-6744-8) contains supplementary material, which is available to authorized users.

## Introduction

Formaldehyde (FA) is a hydrophilic chemical used in antiseptics, adhesives, and lacquers worldwide. Recently, it has been documented that exposure to FA adversely affects human health. The International Agency for Research on Cancer (IARC) classifies FA as a group I compound that is carcinogenic in humans (IARC, 2006). In addition, exposure to FA may cause sick building syndrome and multiple chemical sensitivity (Sahlberg et al., 2013). Garrett et al. (1999) reported that FA exposure can be linked to the prevalence of allergic diseases. The United States Environmental Protection Agency (U.S. EPA) set the maximum FA level in indoor air at 0.1 ppm (U.S. EPA, 1997). In addition, the guideline FA value established by the World Health Organization (WHO 1989), and the Ministry of Health, Labour, and Welfare of Japan (MHLW 2002) is 0.08 ppm.

FA is frequently used to embalm human cadavers used in gross anatomy laboratories for education of medical and dental students. Dissection of embalmed cadavers generally proceeds (limited to thoracoabdominal) as follows: incision of the skin, removal of subcutaneous adipose tissues, and opening of the thoracoabdominal cavity. Recent studies have indicated that the vapor level of FA released from embalmed cadavers increases during dissection, leading to increased FA levels in dissection laboratories during gross anatomy sessions and resulting in students and lecturers suffering itching of the eyes and increased secretion of nasal mucus (Hisamitsu et al., 2011; Mori et al., 2013). In previous studies, we evaluated FA levels in gross anatomy laboratories (Ohmichi et al., 2004, 2006a, 2007). Our data indicated that students could be exposed to 0.89–1.08 ppm of FA during laboratory sessions (Ohmichi et al., 2006a). In addition, total blood IgE levels did not increase significantly and IgE specific to FA was not detected during laboratory sessions (Ohmichi et al., 2006b).

It has been reported that the FA level in dissection rooms changes during laboratory sessions. Our previous research also indicated that FA levels differ between anatomical sessions. Our data showed room averages of FA vapor levels of 0.45, 0.38, and 0.68 ppm during the 4th, 10th, and 18th laboratory sessions, respectively (Ohmichi et al., 2006a). Takayanagi et al. (2007) reported breathing-zone FA levels of 0.50 ppm (decortication of the ventral trunk), 2.00 ppm (decortication of the superficial muscles of the dorsal trunk), 2.64 ppm (digestive tract), 3.04 ppm (posterior abdominal wall), and 1.92 ppm (decortication of the upper and lower extremities) during the dissection process. Kunugita et al. (2004) reported FA levels of 94, 605, 1386, and 986 ppb for the 1st, 2nd, 3rd, and 4th measurements, respectively. From these studies, we hypothesized there are high-risk stages during the dissection process in which the FA vapor level increases dramatically. However, no longitudinal analyses of the release of FA vapor from the thoracoabdominal region of embalmed cadavers have been reported. To identify the high-risk stage(s) with respect to FA vapor exposure, we evaluated the levels of FA vapor released from embalmed cadavers in each dissection stage.

## Materials and methods

### Consent for cadaver use

Informed consent for cadaver use for research and education purposes was acquired from the members of the Whole-Body Donation Registry at Chiba University and from the Chiba Shiragiku Community and their families.

### Embalming

Perfusion fixation was used to embalm cadavers. Briefly, 3 L of preconditioning solution [consisting of 3.3 % pH-A, 10 % Cell Conditioner solution (Champion Company, OH, USA), and 86.7 % water] was injected into each cadaver, and, subsequently, 20 L of preservative-fixative solution [31.1 % masked-form 2A (Japan Tanner Corporation, Osaka, Japan; containing approximately 7.4 % FA), 38.8 % ethanol, 13.8 % glycerol, and 16.3 % water] was added through the femoral artery using a pressure-injection device (Duotronic II, Pierce Companies, TX, USA). The final concentration of FA in the preservative solution was approximately 2.3 %. Blood removal was carried out via the superior sagittal sinus. The embalmed cadavers were encased in storage bags and preserved for approximately 2 years and used for analyses.

### Definition of anatomic stage

**Fig. 1:**
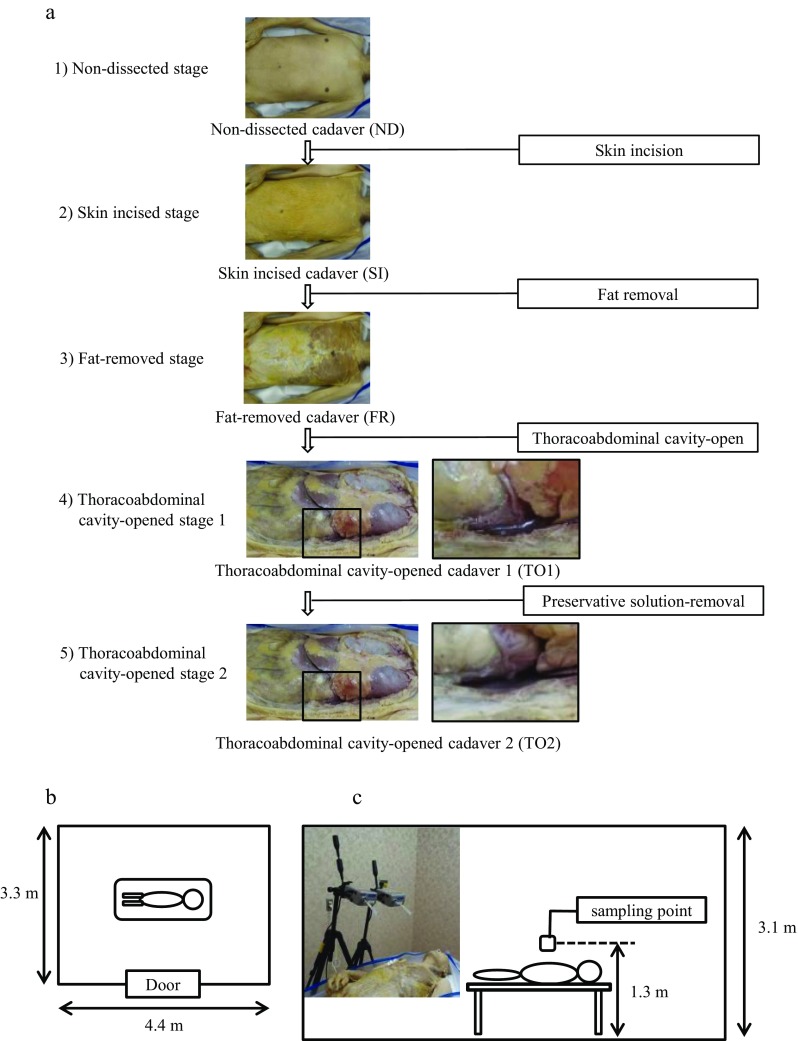
Illustration of the procedure used for evaluation of FA vapor levels. **a** Each dissection stage, showing a dissected cadaver and flowchart for evaluation of FA vapor levels. **b** Diagram of the assessment room. **c** Photograph and scheme of sampler position for capturing FA vapor

Cadaver dissection proceeded as shown in Fig. 1. FA was collected during the following stages: (1) non-dissected (ND) stage, (2) skin-incised (SI) stage, (3) fat-removed (FR) stage, (4) thoracoabdominal cavity–opened stage 1 (TO1, with preservative solution), and (5) thoracoabdominal cavity–opened stage 2 (TO2, without preservative solution) (Fig. 1a). To collect FA vapor released from adipose tissues, subcutaneous adipose tissues were isolated from the cadavers in the SI stage. During each stage, the cadavers and adipose tissues were encased in storage bags until FA evaluation.

### Measurement of FA level

Six cadavers were examined in this study (Table 1). The measurement of FA level was carried out in the assessment room [3.3 m × 4.4 m × 3.1 m (Fig. 1b, c)]. In each dissection stage, storage bags encasing cadavers were transferred into the assessment room and the bag fastener was opened just prior to the analysis. After 30 min, FA was collected using the active sampling method. Briefly, a sampler impregnated with DNPH, Lp DNPH S10, or Lp DNPH H10 (Supelco Inc, MO, USA) was set above the thoracoabdominal region of the cadaver with a dorsal position at a height of 1.3 m from the floor (Fig. 1c). Indoor air was collected using a suction module (MP-∑100-H, Sibata Scientific Technology Ltd, Saitama, Japan) operated at a flow rate of 1 L/min for 30 min to trap FA vapors. Samplers containing trapped FA were stored at −20 °C until analysis. After the sampling was completed, the room was ventilated for 60 min to allow the FA level to return to background prior to the next sampling.

**Table 1:** Cadaver information

Sex・ID	Age (years)	Height (cm)	Weight (kg)	Amount of subcutaneous adipose tissue (thoracoabdominal region) (g)
Male NO.1	85	166.0	79.0	1030
Male NO.2	78	160.0	64.5	1096
Male NO.3	88	150.0	53.5	848
Average ± SD	83.7 ± 4.2	158.7 ± 8.1	65.7 ± 12.8	991.3 ± 128.4
Female NO.1	91	154.0	60.5	2363
Female NO.2	75	154.0	49.0	1787
Female NO.3	83	151.0	36.0	187
Average ± SD	83.0 ± 6.5	153.0 ± 1.7	48.5 ± 12.3	1445.7 ± 1127.4

FA was eluted from the samplers using acetonitrile and analyzed by high-performance liquid chromatography (HPLC). The FA level was calculated as the average value from two samplers and corrected based on indoor temperature and humidity.

### Statistics

Differences in FA vapor levels released from cadavers in each dissection stage were evaluated using the Kruskal-Wallis test. Significance was set as a *P* value of <0.1. Dunn’s test and independent *t* test were used to compare FA levels between cadavers and/or dissected adipose tissues.

## Results

### Level of FA vapor released from embalmed cadavers in each dissection stage

**Fig. 2:**
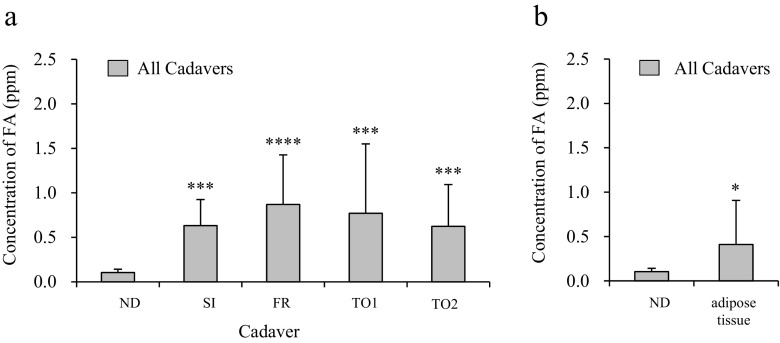
Level of FA vapor released from embalmed cadavers in each dissection stage and released by subcutaneous adipose tissues isolated from the thoracoabdominal region of embalmed cadavers. **a** The significance of differences in FA vapor level between cadavers in each stage was evaluated using the Kruskal-Wallis test (*P* < 0.1). The FA vapor levels from SI, FR, TO1, and TO2 cadavers were compared to that from ND cadaver. Significantly higher FA levels were released from the SI, FR, and both TO cadavers as compared with ND cadavers. *N* = 6 (Male = 3, Female = 3), ***P < 0.01 relative to ND, ****P < 0.001 relative to ND. **b** Data show level of FA vapor released from the subcutaneous adipose tissues of the thoracoabdominal region in embalmed cadavers. The level of FA from adipose tissues was significantly higher than that of ND cadavers. *N* = 6 (Male = 3, Female = 3), *P < 0.1 relative to ND

To clarify the level of FA released in each dissection stage, we determined the levels of FA vapor released from ND, SI, FR, TO1 (before removal of intrathoracoabdominal preservative solution), and TO2 (after removal of intrathoracoabdominal preservative solution) cadavers (Fig. 2a). The vapor level of FA released from the ND, SI, FR, TO1, and TO2 cadavers was 0.10, 0.63, 0.87, 0.77, and 0.62 ppm, respectively. Significantly higher levels of FA vapor were released from cadavers in all dissection stages (i.e., SI, FR, TO1, and TO2) as compared with ND cadavers. Furthermore, the vapor level of FA released from subcutaneous adipose tissues of the thoracoabdominal region was evaluated using adipose tissues isolated from SI cadavers. Our data showed that the subcutaneous adipose tissues released more FA vapor than was released from ND cadavers (Fig. 2b).

### Level of FA vapor released from embalmed male and female cadavers

**Fig. 3:**
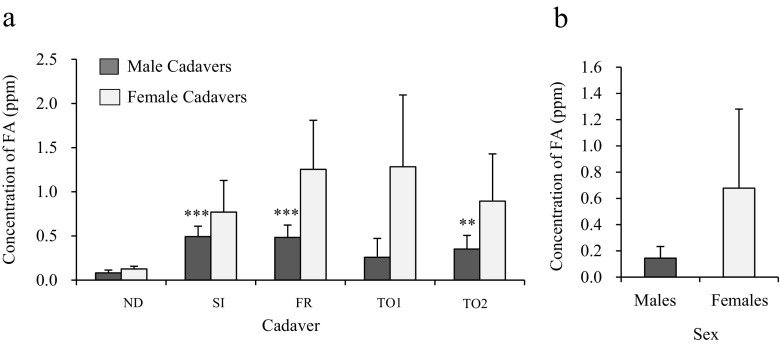
Differences of FA vapor levels released from embalmed cadavers of each dissection stage and released by subcutaneous adipose tissues of the thoracoabdominal region in male and female. **a** The level of FA vapor released from SI, FR, TO1, and TO2 male and female cadavers was compared to that released from ND male and female cadavers. The increase of FA levels was found in all dissected-male and female cadavers as compared to ND. In particular, such increase was significant in SI, FR, and TO2 male cadavers. However, the differences were not significant in female cadavers. *N* = 6 (Male = 3, Female = 3), **P < 0.05 relative to ND, ***P < 0.01 relative to ND. Also, FA vapor levels were compared between male and female cadavers in each dissection stage. Higher levels of FA vapor were released from female cadavers than male cadavers, although the differences were not significant. **b** Comparison of the vapor level of FA released from subcutaneous adipose tissues of the thoracoabdominal region isolated from embalmed male and female cadavers. Higher level of FA vapor was released from female cadavers than male cadavers, but the difference was not significant

The level of FA released from male cadavers in all dissection stages was higher than that released from male ND cadavers (Fig. 3a). The difference in the level of FA released was particularly significant for SI, FR, and TO2 cadavers. In female cadavers, the vapor level of FA released was higher for all dissection stages as compared with ND cadavers, but the differences were not significant (Fig. 3a). In addition, our data showed that the vapor level of released from female cadavers was higher than that released by male cadavers in all dissection stages (Fig. 3a), although the differences were not significant. The vapor level of FA released from subcutaneous adipose tissue was also higher in female cadavers than male cadavers, although the differences were not significant (Fig. 3b).

**Fig. 4:**
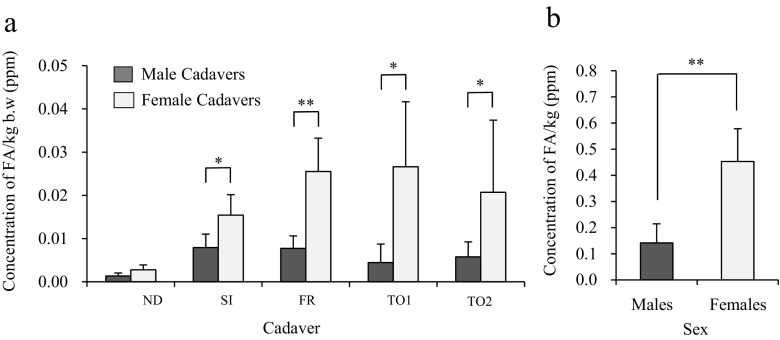
Differences of FA vapor levels released from embalmed cadavers per kilogram body weight of each dissection stage and released by subcutaneous adipose tissues per kilogram of the thoracoabdominal region in male and female. **a** The level of FA released from each embalmed cadaver per kilogram body weight (b.w.) was compared between male and female cadavers in each dissection stage. Higher levels of FA per kilogram b.w. were released from female cadavers than male cadavers. These differences were significant for the SI, FR, TO1, and TO2 stage cadavers. *N* = 6 (Male = 3, Female = 3), *P < 0.1 relative to male cadavers, **P < 0.05 relative to male cadavers. **b** Comparison of the vapor level of FA released per kilogram of adipose tissue of male and female cadavers. Significantly higher levels of FA vapor per kilogram of adipose tissue were released from female cadavers than male cadavers. *N* = 6 (Male = 3, Female = 3), **P < 0.05 relative to subcutaneous adipose tissues from male cadavers

On the other hand, the vapor level of FA released per kilogram body weight (b.w.) was significantly higher for female cadavers than male cadavers except for ND cadavers (Fig. 4a). Also, the level of FA released per kilogram of adipose tissue was significantly higher in female cadavers than male cadavers (Fig. 4b).

## Discussion

In this study, we found that significantly higher levels of FA vapor are released from cadavers at all dissection stages (i.e., SI, FR, TO1, and TO2) as compared with ND cadavers (Fig. 2a). Importantly, the levels of FA vapor released from SI, FR, TO1, and TO2 cadavers (0.63, 0.87, 0.77, and 0.62 ppm) exceeded the maximum value established by the U.S. EPA (0.1 ppm) and the guideline value established by the WHO and MHLW (0.08 ppm). In the official method, the indoor air is collected in each corner and central part of the assessment room and evaluated. Herein, our previous study indicated that the central part has a higher FA level than the corners (Ohmichi et al., 2006a) when cadaver dissection. In the presented study, meanwhile FA level was evaluated at the central part of the assessment room (Fig. 1), we confirmed that FA level in the corner also partially exceeded the maximum value or guideline value (Supplemental Table). On the other hand, no reports have been published regarding the relationship between dissection stage and FA vapor level. Our data suggest that the vapor level of FA released begins to increase after skin incision. The skin thus appears to prevent the release of FA vapor from embalmed cadavers. Once the skin is removed, the vapor level of FA released increases markedly. This result indicates that dissectors should take precautions to minimize FA exposure after skin incision, as exposure to high levels of FA can adversely affect human health (Sarigiannis et al., 2011).

Our data show that male cadavers in all dissection stages release more FA vapor than ND cadavers. In particular, significantly higher levels were released from SI, FR, and TO2 cadavers as compared with ND cadavers (Fig. 3a). In contrast, although female cadavers at all dissection stages also released more FA vapor than ND cadavers, the differences were not significant (Fig. 3a). These data demonstrate that the pattern of FA vapor release in each dissection stage differs between male and female cadavers. For example, FA vapor levels decreased after the thoracoabdominal cavity was opened in male cadavers, but this was not the case in female cadavers. In addition, our results indicated that female cadavers released higher levels of FA vapor than male cadavers in each dissection stage (Fig. 3a), although the differences were not significant. It has been reported that exposure to more than 1 ppm of FA causes irritation of the eyes and upper respiratory tract (Weber-Tschopp et al. 1977; Kulle et al., 1987). Interestingly, the level of FA released per kilogram b.w. by female cadavers was also higher than that of male cadavers (Fig. 4a) and the difference was significant for SI, FR, TO1, and TO2 cadavers. No reports have been published regarding differences in the vapor level of FA released by male and female cadavers. Although differences were observed in the present study, we could not evaluate the underlying mechanism. Importantly, however, our data could indicate that female cadavers present a higher FA exposure risk to dissectors than do male cadavers. Further detailed research will be necessary in order to elucidate the reason why male and female cadavers differ with respect to release of FA vapor and to reduce the risks associated with the higher levels of FA released by female cadavers.

In addition, we confirmed that subcutaneous adipose tissues isolated from SI cadavers release FA vapor. The vapor level of FA released from the adipose tissues was approximately 2–3 times greater than that released by ND cadavers (Fig. 2b). The vapor level of FA released by subcutaneous adipose tissues isolated from female cadavers was higher than that released by the tissues of male cadavers (Fig. 3b). Interestingly, a significant difference was observed between male and female cadavers with respect to the vapor level of FA released per kilogram of subcutaneous adipose tissue (Fig. 4b). These data suggest that the subcutaneous adipose tissue is one of the emitting sources of FA in embalmed cadavers. Although the reasons for these differences between male and female cadavers are currently unknown, recent studies have revealed differences in subcutaneous adipose tissues in males and females. For example, Danilo et al. (2014) reported that the amount of lactase and glycerol produced by subcutaneous adipocytes differs between males and females. Casabiell et al. (1998) reported that female subcutaneous adipocytes secrete higher levels of leptin than do male adipocytes. Thus, various sex-specific differences in the characteristics and properties of subcutaneous adipose tissues may contribute to the differences in the vapor level of FA released by male and female cadavers.

The data from the present study show that (1) the vapor level of FA released by cadavers in all dissection stages is markedly higher as compared with ND cadavers, (2) female cadavers release higher levels of FA vapor than do male cadavers, (3) subcutaneous adipose tissues release FA vapor, and (4) female cadavers release higher levels of FA vapor from the subcutaneous adipose tissues than do male cadavers. We present data demonstrating the pattern of FA vapor release from dissected embalmed cadavers and reveal in part the mechanism through which FA vapor is released from embalmed cadavers. Our data showing a marked increase in FA vapor release following the skin-incision step of the dissection process indicate that intact skin plays an important role in preventing FA release. Moreover, our results indicate that the subcutaneous adipose tissue is one of the emitting sources of FA in embalmed cadavers. We could not clarify the mechanism underlying differences in the level of FA released by male and female cadavers in this study, as a variety of factors may be involved. Further studies will be needed to provide a more complete understanding of the mechanisms controlling FA vapor release from cadavers.

## Electronic supplementary material

Below is the link to the electronic supplementary material.

Table S1 The levels of FA vapor in corner of the assessment room. (DOCX 23 kb)
