# Supplementary material for: Levels of formaldehyde vapor released from embalmed cadavers in each dissection stage
Source: Environ Sci Pollut Res Int. 2016 May 6;23:16176–82. doi: 10.1007/s11356-016-6744-8 (PMC4975760; doi:10.1007/s11356-016-6744-8)
Supplement: Supplementary file 1 — The levels of FA vapor in corner of the assessment room. (DOCX 23 kb) [file 11356_2016_6744_MOESM1_ESM.docx]

**Supplemental Table. The levels of FA vapor in corner of the assessment room**

|  |  | ND | SI | FR | TO1 | TO2 |
| --- | --- | --- | --- | --- | --- | --- |
| Male (N=3) | Ave ± SD | 0.05 ± 0.02 | **0.14** ± 0.03 | **0.17** ± 0.04 | **0.16** ± 0.06 | **0.24** ± 0.01 |
| Female (N=3) | Ave ± SD | 0.06 ± 0.00 | **0.30** ± 0.07 | **0.33** ± 0.04 | **0.43** ± 0.13 | **0.45** ± 0.03 |
| all (N=6) | Ave ± SD | 0.05 ± 0.01 | **0.22** ± 0.10 | **0.25** ± 0.09 | **0.30** ± 0.17 | **0.35** ± 0.11 |

( ppm )

Bold numbers indicate a value that exceeds the guideline value established by the WHO and MHLWJ (0.08 ppm)
